# Knowledge of gendered needs among the planners and policy makers for prevention of NCDs in Bangladesh: a qualitative exploration

**DOI:** 10.1186/s12939-024-02186-4

**Published:** 2024-05-27

**Authors:** Sadika Akhter, Mohammed Kamruzzaman, Iqbal Anwar, Mahmuda Shaila Banu, Daniel D Reidpath, Adrian J Cameron

**Affiliations:** 1 School of Health and Social Development, Deakin University, 221 Burwood Hwy, Burwood, Melbourne, VIC-3125 Australia; 2 Health Systems and Population Studies Division, icddr,b 68 Shaheed Tajuddin Ahmed Sarani, Mohakhali, Dhaka, 1212 Bangladesh; 3 World Health Organization, Dhaka, 1212 Bangladesh; 4 School of Education, RMIT University, Melbourne, VIC-3000 Australia; 5 Institute for Global Health and Development, Queen Margaret University, Edinburgh, Scotland

## Abstract

**Background:**

Globally, non-communicable diseases (NCDs) are increasingly the primary cause of mortality and morbidity among women. Like many developing countries, Bangladesh also faces a growing burden of NCDs. The “Multisectoral Action Plan for Prevention and Control of Non-communicable Diseases, 2018–2025” signifies Bangladesh’s commitment to comprehensively combating the rising burden of NCDs. This study investigates the perceptions of those involved in developing the action plan and if/how a gender lens was incorporated into its implementation.

**Methods:**

In-depth interviews were conducted with 25 key individuals involved in a high-level committee to develop and implement Bangladesh’s multisectoral action plan to address the burden of NCDs. Data were collected between July and November 2021, and thematic analysis was conducted.

**Results:**

The findings revealed that interviewees believed the multisectoral action plan adopted a population-wide approach without considering gender-specific needs. This study presents the explanations for this inattention under five themes: (1) A population-level approach to NCD prevention; (2) Understanding women’s health beyond reproductive health; (3) Absence of gender-specific programs; (4) Lack of consideration of gender constraints on physical activity; and (5) Lack of collaborative efforts to address NCDs beyond the health ministry.

**Conclusion:**

In conclusion, governments in countries like Bangladesh can develop more effective strategies to reduce the disease burden of NCDs among women by recognizing and addressing the gendered nature of preventive health. This can be achieved by promoting gender-responsive research, programs, and policy initiatives that consider women’s specific health concerns, ultimately leading to better health outcomes for all.

## Background

Non-communicable diseases (NCDs) have become a significant public health issue in both developed and developing countries [1]. These chronic diseases, which include cardiovascular diseases, cancer, chronic respiratory diseases, and type 2 diabetes, among others, are responsible for 80% of deaths worldwide and 77% of all deaths in low and middle-income countries (LMICs) [2]. NCDs affect the lives and life expectancies of both men and women. For women in LMICs, NCDs are an increasingly significant cause of morbidity and mortality and are rivaling maternal health and undernutrition in their contribution to the burden of disease [2, 3]. NCD risk factors that are increasing in prevalence in recent decades include hypertension, overweight and obesity, pre-diabetes, and dietary behaviours, including high sodium intake and low fruit, vegetable, and whole grain consumption [4–7].

Gender was acknowledged as a significant driver of women’s reproductive health at the International Conference on Population and Development (ICPD), Cairo, 1994, and the Fourth World Conference on Women, Beijing [8–10]. Following the Cairo Declaration, research on issues relating to women’s reproductive health increased and was prioritized by funding agencies, while research into the high burden of NCDs among women was neglected in comparison [11, 12]. This was even though two out of every three women die from NCDs [11]. The WHO (2018) have highlighted the range of economic and social factors that lead to women (particularly those in resource-poor settings) being disproportionately affected by NCDs, including limited access to quality health services and gender norms [13].

Universal Health Coverage (UHC) is a global goal that aims to ensure that all individuals and communities can access essential health services without facing financial hardship [14]. It is built on the equity principle that everyone, regardless of their economic status, should have access to quality healthcare services when needed [14, 15]. Achieving Universal Health Coverage (UHC) for women can help reduce their disadvantages and the high incidence of NCDs. It has the potential to provide women with better access to quality health services and to address the range of related economic and social factors that contribute to gender-based NCD disparities [16, 17].

Bangladesh faces a double burden of communicable and non-communicable diseases, with NCDs emerging as an urgent public health challenge [18]. Approximately 75% of the population is living with multiple NCD risk factors, and 67% of all deaths in the country are from cardiovascular diseases, type 2 diabetes, cancers, and chronic respiratory conditions [19]. In Bangladesh, the prevalence of NCDs will be greater among women (33.2%) than men (24%) by 2030 [19]. In particular, the prevalence of hypertension, obesity, and cardiovascular diseases has been found to be higher among women than in men (24% vs. 18%; 33% vs. 18% and 11% vs. 9%, respectively) [19].

A significant contributing factor to the higher prevalence of NCDs among women compared to men, is the deep-rooted gender inequalities and unequal power relations present in much of Bangladeshi society [20]. Although women’s economic autonomy plays a significant role in determining their health, patriarchal cultural norms and practices constrain women to own land, making decisions, and exercise economic independence [21]. This consequently restricts their access to healthcare services, their capacity to choose what food to buy, and their ability to obtain information regarding the management and prevention of non-communicable diseases (NCDs) [22, 23].

Gender-insensitive health policies and programs fail to adequately address the unique health needs and challenges faced by women in Bangladesh, including NCD prevention and management [24]. Government funding and resources are often disproportionately allocated to communicable disease control and maternal health, neglecting the growing burden of NCDs among women [25]. A lack of political will to challenge traditional gender norms and promote gender equity further can also further perpetuate the disparities in NCD prevalence and outcomes among women [26].

The multisectoral action plan for NCD prevention in Bangladesh implemented since 2018 specifically recognized the gender-specific burden of NCDs and the need for a gender-sensitive approach to preventing and controlling NCDs [27]. The action plan identifies gender as a key determinant of health. It acknowledges that women in Bangladesh face particular challenges related to NCDs, such as limited access to healthcare, limited control over resources, and cultural barriers that prevent them from adopting healthy lifestyles [27]. It is unclear, however, if the implementation of the action plan has addressed these gender concerns in practice. This study uses a qualitative design to explore the perspectives of those involved with developing and implementing the multisectoral action plan regarding how it has addressed gender considerations in preventing NCDs.

## Methods

### Study design, setting, and sampling

We conducted a qualitative exploratory study using purposive sampling [28]. The Bangladesh Ministry of Health and Family Welfare prepared a comprehensive list of individuals involved in the High-Level Technical Committee (HLTC) for preventing and controlling non-communicable diseases (NCDs) in Bangladesh since 2018, selected from different government ministries, NGOs, and academic institutions [27]. These people (*n* = 25) provided technical guidance and support to the government in developing, implementing, monitoring, and reviewing strategies, policies, and programs related to the prevention and control of NCDs in Bangladesh.

### Data collection

The study used a standard semi-structured interview guide developed based on a literature review and discussion with the study team members [29]. The interview guide covered various topics, including interviewees’ involvement in the planning and implementation of the multisectoral action plan. It further explored the participants’ knowledge and ability to promote gender issues in policies and programs related to NCDs. All interviews were recorded with a digital recorder and transcribed by SA. The open-ended nature of the interview questions helped to generate free discussions with the study participants.

Further, the researchers used probing techniques to gain deeper insights into the study participants’ knowledge and experience. The interview guide was pretested, and although no significant challenges were reported during pretesting, minor language editing was done to revise the interview guide. Data collection took place between July and November 2021. The interviews were conducted face to face. SA and MK conducted interviews, and both have a background in anthropology and extensive experience conducting similar interviews. The interviews were conducted in the participant’s native language, Bengali. The length of the interviews was, on average, 40–45 min. All interviews were digitally recorded using a digital recorder with the written consent of the participants. The researchers conducted the interviews in an environment where no one else was present to ensure data privacy and avoid influencing the responses. No repeat interviews were conducted.

### Data analysis

The study employed a thematic analysis with a mixed deductive and inductive coding schema to code the transcripts [29]. Deductive coding was performed using pre-determined codes derived from the literature on social determinants of Health, NCDs, and gender [30, 31]. New codes generated from the interviews were added inductively.

The coding and data analysis were conducted concurrently during data collection. Two authors (SA and MK) read and re-read full transcripts several times to fully understand the data and confirm a high level of data absorption during data coding. The transcripts were coded using a combination of sentence-level and paragraph-level units according to the participants’ responses. This coding approach allowed for a detailed investigation of individual sentences and the broader context provided by entire paragraphs. The purpose was to portray and classify meaningful patterns, themes, or concepts within the participants’ responses to understand data comprehensively. Data saturation was reached after coding the 20th interview, with no emerging topics appearing past this point. However, all data were coded to ensure that all participants’ views were included in subsequent analyses. A unique ID number for the transcripts was assigned to maintain privacy and anonymity, with a separate file linking this ID number to identifying information. NVivo11 software was used to manage interview transcripts and coding.

## Results

### Profile of the study participants

A total of 25 interviews were conducted. Five study participants refused to participate, mentioning scheduling conflicts and busy agendas. The study participants, (2 women and 23 men) were from the Ministry of Health and Family Welfare (5), Ministry of Planning (2), Ministry of Industries (2), Ministry of Food (3), Ministry of Youth and Sports (1), and the Ministry of Women and Children Affairs (2). Five participants were interviewed from national and international NGOs/INGOs, three from academic institutes, and two representatives from the Consumers Association of Bangladesh who were also part of the HLTC (Table 1). The interviews were conducted to explore participant’s perspectives on the deimplementing the multisectoral action plan, integrating a gender lens in preventing non-communicable diseases (NCDs).

**Table 1:** Characteristics of study participants

Variable	*N* = 25
**Sex**	
Male	23
Female	2
**Affiliation**	
Government officials	15
NGOs/INGOs representatives	5
Academics/Consumers Association of Bangladesh	5

**Fig. 1:**
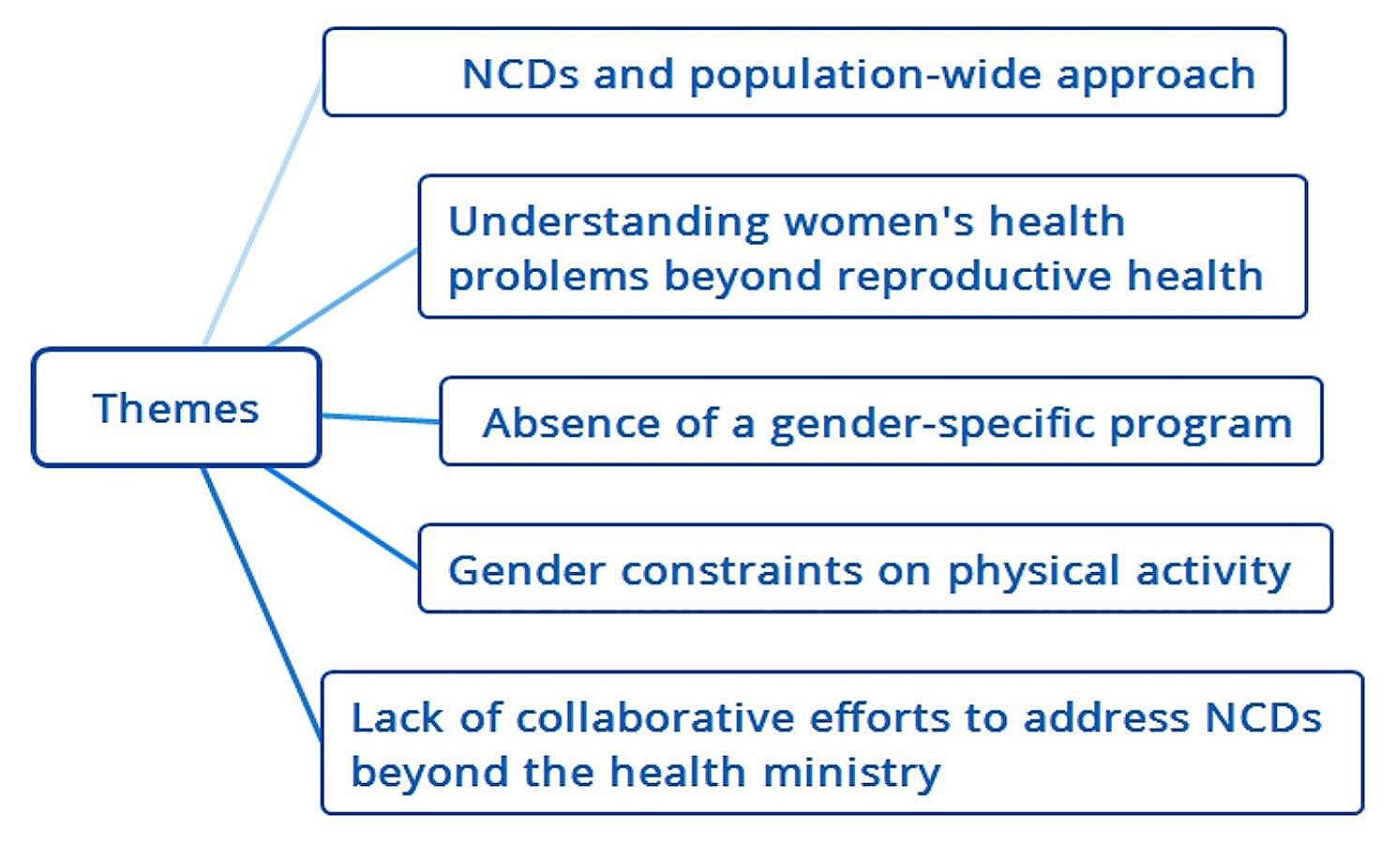
The themes resulting from the analysis of interviews with 25 individuals involved in the development and implementation of the NCD action plan in Bangladesh

The study findings are presented in five main themes: NCDs and population-wide approach; Understanding women’s health problems beyond reproductive Health; Absence of gender-specific programs; gender constraints on physical activity; and lack of collaborative efforts to address NCDs beyond the health ministry (Fig. 1).

### Theme 1: NCDs and population-wide approach

Concerning implementing the population-wide strategy intended to tackle the issue of non-communicable diseases (NCDs), participants emphasized that a shift from communicable to non-communicable diseases was evident, particularly in LMICs. They further added that NCDs have now emerged as the primary cause of mortality and disability in Bangladesh. One respondent indicated,

*We have adopted the whole government’s and society’s efforts for health promotion to prevent NCD. So that the disadvantaged people, the poor people, get an advantage. One of the key goals of this approach is to ensure equity, which aims to reduce health disparities and ensure that vulnerable populations receive the necessary support and opportunities for the prevention and management of NCDs.*

The discussion with the respondents revealed that a whole-of-society approach had created challenges in addressing the gender-specific needs of NCDs in Bangladesh. They further explained that systematic data collection and analysis was not stratified by gender (or age) to inform and implement equity-focused policy and programs in the country. As an example, study participants noted that no data is recorded for the number of women with gestational diabetes and high blood pressure during pregnancy. They stated that the country needs better systems to ensure accurate and relevant data is available for making decisions about NCD prevention and management for women in Bangladesh.

### Theme 2: Understanding women’s health problems beyond reproductive health

All interviewees stated that Bangladesh has made significant efforts to improve women’s health, predominantly focusing on their sexual and reproductive health, which has significantly reduced maternal mortality. They added that since the introduction of the Millennium Development Goals (MDGs) in 2000, the country and donors have worked together to address the significant disease burden related to maternal health. Most of Bangladesh’s health research, programs, and activities still focus on maternal and child health and undernutrition. The participants in the interviews emphasized that it is fundamental to understand that addressing women’s health needs should go beyond reproductive health, as non-communicable diseases (NCDs) are now a more significant threat to women’s lives in the country than any other disease.

In relation to this, a respondent from an international organization said,

*Our health policymakers have a serious lack of knowledge and understanding to address the problem of NCDs with considerations for gender. We do not have training on gender to understand health problems from a gender perspective. The lack of knowledge among policymakers about the issue of gender has created a significant barrier for us to move forward.*

According to all interviewees, research funding for developing and implementing health interventions in Bangladesh heavily depends on foreign donors. The allocation of funding is mainly based on the donors’ priorities and preferences, leading to a donor-driven approach to health programming. Instead of supporting NCD prevention and women’s health, the donors are still interested in funding sexual and reproductive health programs and research. They added that the donors and government should design more capacity-building programs, training, and workshops for the policymakers to build their capacity. This would ensure a comprehensive understanding of how gender influences NCDs and enable local policymakers to negotiate with donors to secure more funding. Gender experts are always invited to attend meetings when there is an issue of violence against women, but they are hardly ever invited to provide their expert opinions about NCDs and gender.

One participant stated, “*I attended some high-level meetings about addressing NCDs in the country but never heard anybody in the meeting talking about understating the NCDs with a gender perspective. I am always invited to attend these meetings, but I cannot provide any input related to gender and NCDs as I do not have any expertise in this field.”*

The participants reported that among the international organizations, the WHO played a crucial role in providing technical guidance and mobilizing resources to support the country in addressing the growing burden of NCD-related health challenges, but more donors need to get involved in this issue to address the problem.

Another participant said, “*We generally understand gender means violence against women, child marriage, maternal health, and reproductive health. The WHO always supports the government in tackling the burden of NCDs, but it is crucial that other donors should prioritize their funding to address the problem of NCDs with a focus of gender in Bangladesh*.”

### Theme 3: Absence of a gender-specific programs

The interviewees noted that the prevalence of smoking among women is less than among men in Bangladesh. The government is increasing the tobacco price to lower the smoking rate among men. But more women than men use smokeless tobacco in the country. They further explained that the country adopted the WHO-recommended target to reduce tobacco use by 30% by 2025. The participants said the target is not gender inclusive, and the voluntary target to reduce tobacco use is not gender specific. With many women using smokeless tobacco, meeting the WHO target by only increasing the tax and price of tobacco for smoking will be a big challenge for the country.

One respondent explained,

*Our society views tobacco smoking as an anticipated male custom. However, the rate of female smokeless tobacco use is higher than that of males.* Moreover, there is a rising trend of cigarette smoking among women, attributing it to factors such as fashion and the association of smoking with social and economic status. *If we try to reduce the use of tobacco only targeting the men, we are potentially excluding the women who are using smokeless tobacco more than men.*

In these discussions, the respondents also noted that the country agreed to a 30% reduction of salt intake in the population by 2025 and the WHO target of < 5 g salt consumed per day. They further explained that promoting population-wide interventions to reduce salt intake will likely significantly reduce preventable deaths from CVDs. The interviewees further highlighted a gap in educational initiatives. They pointed out that women play a vital role in Bangladesh at the household level, and there is no educational program targeting women to educate and empower them with the knowledge to reduce salt consumption at the household level. The NCD program manager provided the following explanation regarding the absence of a clear approach concerning gender considerations in salt policy,

*We do not have data on salt intake at the population level. In our culture, women play the main role in the household, cooking and serving food. We did not include a gender perspective in our multisectoral action plan targeting women, in particular, to educate them to reduce the use of salt for cooking and serving.*

### Theme 4: Gender constraints on physical activity

The study’s participants indicated that Dhaka, the capital of Bangladesh, is experiencing rapid urbanization. They explained that the environment of Dhaka creates challenges for women, particularly young and adolescent girls, who face barriers to engaging in physical activity. The study participants reported that women do not feel safe when going out for physical activity, and women often encounter teasing, bullying, and negative comments when exercising. This creates a threatening and discouraging atmosphere that hinders their participation in physical activity.

Many study participants also reported that physical activity, especially among women in urban areas, is limited due to their gender roles within households, where women are primarily responsible for household chores, leaving them with limited time and opportunity for physical exercise. The study participants suggested that the accessibility and availability of urban infrastructural facilities and public places play a significant role in determining women’s ability to engage in physical activity.

One respondent commented,

*Obesity and diabetes are increasing among women in our country. Urban women mostly spend a sedentary life. Women do not have many opportunities to perform physical activity, especially in Dhaka. Women experience abusive behavior when they go outside for physical activity. I would say the city is not yet women-friendly. I don’t see our policies addressing these issues to make the city more women-friendly so that all women, girls, and adolescents can participate in physical activities.*

Another respondent said,

*Obesity among urban women and adolescents is increasing. But we hardly see that these obese adolescents are going out for physical activity as a part of a healthy lifestyle. They are not going out because the public spaces are not safe and friendly for them. We need to address these issues through policies and programs through a gender approach.*

### Theme 5: Lack of collaborative efforts to address NCDs beyond the health ministry

All study participants agreed that the Ministry of Health cannot shoulder the burden of NCDs alone. Instead, a collaborative and cross-sectoral approach is required to tackle the growing challenge of NCDs in Bangladesh effectively. Respondents emphasized the role of municipalities in taking responsibility and adopting a citizen-friendly approach, particularly with a focus on the needs of women. They stressed the importance of creating safe and supportive urban spaces, encouraging physical activity, and facilitating healthy living for all residents.

Furthermore, the study participants stressed the importance of inter-ministerial coordination and collaboration to pool resources, expertise, and knowledge from diverse sectors, leading to more comprehensive and effective interventions. The interviewees expressed their opinion that there is currently a lack of mechanisms for regular communication, information sharing, and joint decision-making among ministries so that they can work together towards a common goal of addressing NCDs.

One respondent said, *“Addressing NCDs goes beyond the scope of a single ministry, but not all ministries are actively engaged or working collaboratively in addressing NCDs. When any issue comes about health, another ministry tends to shoulder its responsibility to the health ministry. As a result, we are not progressing to achieve our goals.”*

All of the stakeholders mentioned improving coordination among different ministries and donors to align their funding priorities and include a gender lens for NCD control in the country. They suggested encouraging donors to integrate gender considerations in their funding criteria and supporting gender-focused research and capacity building of policymakers to understand the problem of NCDs and program implementation from a gender-sensitive standpoint.

## Discussion

Equity is a fundamental principle in public health policy, aiming to ensure the fair and just distribution of health opportunities, resources, and outcomes among different population groups [32, 33]. Exploring equity concerning the burden of NCDs in Bangladesh within a major whole-of-government initiative, our study presents a valuable gender perspective on how the policy goal of NCD prevention can be successfully pursued amidst other competing policy objectives. This study aimed to assess HLTC members’ perceptions of NCD prevention practices in Bangladesh, with a focus on examining gender in these efforts.

Similar to previous studies on NCDs and gender, we have found several key factors that influence the understanding of the burden of NCDs from a gender perspective [34, 35]. The factors identified in this study include policymakers’ lack of knowledge and expertise about the gender perspective in NCD prevention. The study participants recognized that it is important to design gender-sensitive health promotion activities to address the NCD risk factors of women in the country. They added that cultural and social norms often limit women’s access to physical activity opportunities. Socio-cultural factors, such as traditional gender roles, often assign women the responsibility of preparing meals and managing household chores. Despite this, women often have limited control over food choices and prioritize the preparation of meals that are expected by their family rather than those that promote healthier dietary habits in Bangladesh [20, 21]. Participants emphasized capacity building of policymakers around understanding gender-specific issues related to NCD prevention, as well as training for healthcare providers to be sensitive to gender issues and to provide care designed to cater to the needs of women.

Our findings shed light on the challenges and opportunities within the current multisectoral action plan aimed at controlling NCDs, specifically focusing on understanding and addressing gender disparities. The study reveals that policymakers’ lack of knowledge and expertise poses a significant hurdle in comprehending the gendered needs associated with NCDs. The absence of sex- and age-disaggregated data on the prevalence and incidence of NCDs hinders the ability to identify and address gender disparities in NCDs effectively. Such data is crucial for understanding the unique risk factors, prevalence, and impact of NCDs among different gender groups.

Our findings align with previous research about knowledge of the intersection of gender and NCDs, recognizing that this is an important factor in effectively setting priorities [35, 36]. The influence of donors in setting the agenda and the role of government policies were also considered constraints in addressing NCDs with a gender lens. Additionally, participants’ engagement and the utilization of research evidence were identified as crucial components of the priority-setting process [25, 37].

The complex interplay between external donors, governmental policies, and the gendered dimensions of NCDs is a theme identified in previous scholarly work [25]. For instance, Shiffman (2006) highlighted the need for a nuanced understanding of the impact of external influences, such as donor priorities, on shaping health agendas [36]. Other studies have emphasized the constraints imposed by governmental policies in incorporating gender perspectives into health initiatives [38, 39]. Furthermore, our research emphasizes the important role of inclusive engagement and evidence-based research in the priority-setting process [39, 40]. This aligns with the broader literature on participatory approaches and evidence-informed decision-making in health policy formulation [40, 41].

Importantly, our study included only two women out of 25 members on the HLTC, emphasizing the need to include a sufficient number of female gender experts in technical committees like the HLTC. Women with substantial practical knowledge and experience in gender-related issues within their communities, even if they lack formal expertise relating to the specific topic, could also be valuable additions to such committees. This inclusive approach would ensure a broader spectrum of perspectives and insights, enhancing the responsiveness of health policies to gender-specific needs and challenges.

In synthesizing our findings with the existing literature, our study contributes to the evolving discourse on the complex dynamics of prioritizing gender-sensitive strategies for addressing NCDs. The insights gained from this research provide a foundation for future interventions and policies to foster a holistic and practical approach to NCDs that incorporates gender considerations [41].

Based on the study findings, the following actions are recommended to improve the NCD prevention priority-setting process and address the challenges identified.

- Develop targeted educational programs to enhance policymakers’ understanding of gender-specific issues in NCD prevention.
- Promote the design and implementation of gender-sensitive health promotion activities, clearly targeting NCD risk factors among women in Bangladesh.
- Prioritize the collection of sex- and age-disaggregated data to understand better and address gender disparities in NCDs, providing a foundation for evidence-based interventions.
- Engage in advocacy efforts to influence governmental policies and donor priorities, ensuring that gender perspectives are integrated into the agenda for addressing NCDs.
- Facilitate increased collaboration among ministries to promote inclusive engagement and evidence-based research to inform a more effective and holistic approach to NCD prevention in Bangladesh.
- Ensure greater representation of women, including female gender experts in technical committees such as the HLTC.

The study’s findings stressed the collective responsibility of addressing non-communicable diseases (NCDs). It was agreed that the Ministry of Health could not shoulder the burden of NCDs alone. Instead, a collaborative and cross-sectoral approach is required to tackle the growing challenge of NCDs in Bangladesh effectively. Overall, while external donor funding plays a vital role in supporting health interventions, it is crucial to maintain a balanced approach that considers both donor priorities and local needs. The country could effectively and sustainably address the growing challenges of NCDs’ by encouraging greater collaboration, strengthening policymakers’ decision-making capacity, and diversifying funding sources.

### Limitations and strengths

Despite the inherent limitations of qualitative research on the generalizability of the study findings, this study presents significant findings for the priority-setting process for NCDs with considerations for gender, which could be pertinent to other similar settings. A strength of the study is that it was based on data from purposefully recruited participants involved in policy decisions for health in Bangladesh at the national level. Such participants possess firsthand knowledge, expertise, and insights into the complexities of health policy formulation and implementation. Their involvement ensures that the study’s findings are grounded in real-world experiences and are directly relevant to the context of Bangladesh’s health policy landscape.

## Conclusion

This study has emphasized the importance of gender when developing and implementing the multisectoral action plan for addressing the emerging problem of NCDs in Bangladesh. A general population-wide approach to NCD prevention has meant many gender-specific needs have been neglected, and the issue of equity, which means the fair and just distribution of health opportunities, resources, and outcomes among diverse population groups, has not been addressed. Our study acknowledges the limitations of a population-wide strategy. It is crucial to recognize that a one-size-fits-all approach may inadvertently leave certain groups behind, failing to meet their specific needs. Therefore, our findings emphasize the necessity of targeted actions to address the diverse challenges different population groups face.

Policymakers must have the knowledge and expertise to design and implement gender-sensitive health promotion activities. This includes capacity building for healthcare providers and promoting sensitivity to gender issues to address the unique needs of women.

In conclusion, our study shows that while a population-wide perspective is integral to tackling the overall burden of NCDs, it must be complemented by targeted actions that address the unique needs and challenges faced by specific demographic groups, and in the case of Bangladesh, women in particular. By fostering collaboration, strengthening decision-making capacity among policymakers, and diversifying funding sources, Bangladesh can address the increasing burden of NCDs with a commitment to health equity and improved outcomes for all.

## Data Availability

No datasets were generated or analysed during the current study.

## Abbreviations

HLTC: High-Level Technical Committee
NCD: Non-communicable disease
UHC: Universal Health Coverage
