# Correction: Knowledge of gendered needs among the planners and policy makers for prevention of NCDs in Bangladesh: a qualitative exploration

**DOI:** 10.1186/s12939-024-02210-7

**Published:** 2024-07-01

**Authors:** Sadika Akhter, Mohammed Kamruzzaman, Iqbal Anwar, Mahmuda Shaila Banu, Daniel D Reidpath, Adrian J Cameron

**Affiliations:** 1 School of Health and Social Development, Deakin University, 221 Burwood Hwy, Burwood, Melbourne, VIC-3125 Australia; 2 Health Systems and Population Studies Division, icddr,b 68 Shaheed Tajuddin Ahmed Sarani, Mohakhali, Dhaka, 1212 Bangladesh; 3 World Health Organization, Dhaka, 1212 Bangladesh; 4 School of Education, RMIT University, Melbourne, VIC-3000 Australia; 5 Institute for Global Health and Development, Queen Margaret University, Edinburgh, Scotland

**Correction: International Journal for Equity in Health (2024) 23:110**

10.1186/s12939-024-02186-4

After publication of this article, the authors reported that the last author (Cameron) was incorrectly linked to affiliation 2, but he should have been linked to affiliation 1. Moreover, the author name Mahmuda Shaila Banu was incorrectly written as Mahmud Shaila Banu.

The original article has been corrected.

